# Different Treatment Modalities of Oral Lichen Planus—A Narrative Review

**DOI:** 10.3390/dj11010026

**Published:** 2023-01-12

**Authors:** Ana Andabak-Rogulj, Ema Vindiš, Lorena Horvat Aleksijević, Ivana Škrinjar, Danica Vidović Juras, Anastazija Aščić, Božana Lončar Brzak

**Affiliations:** 1 Department of Oral Medicine, School of Dental Medicine, University of Zagreb, 10000 Zagreb, Croatia; 2 Department of Oral Medicine, School of Dental Medicine, University of Zagreb, Clinical Hospital Zagreb, 10000 Zagreb, Croatia; 3 Dental Practice at Healthcare Center Ormož, 2270 Ormož, Slovenia; 4 Faculty of Dental Medicine and Health, University of Osijek, 31000 Osijek, Croatia; 5 Private Dental Practice, 31000 Osijek, Croatia

**Keywords:** oral lichen planus, chronic disease, therapeutics, adverse effects

## Abstract

Oral lichen planus (OLP) is a chronic inflammatory disease of unknown etiology which affects the oral mucosa. OLP varies in its clinical features from a reticular form that is, in most cases, asymptomatic, to atrophic–erosive, and is accompanied by symptoms of burning sensation and pain followed by difficulty in eating. Given the fact that OLP is a disease of unknown etiology, the treatment is symptomatic and involves suppressing the signs and symptoms of the disease using various topical and systemic drugs. The first line of therapy for treating symptomatic OLP is topical corticosteroids, whereas systemic corticosteroids are used for treating persistent lesions that do not respond to local treatment. However, the lack of convincing evidence on the efficacy of previous therapies, including topical corticosteroids, and numerous side effects that have appeared over recent years has resulted in the emergence and development of new therapeutic options. Some of the therapies mentioned are tacrolimus, efalizumab, dapson, interferon, retinoic acid, photochemotherapy with psoralen and ultraviolet A rays (PUVA), aloe vera, antimalarials, antibiotics and others. These therapies only partially meet the properties of efficacy and safety of use, thus justifying the continuous search and testing of new treatment methods.

## 1. Introduction

Lichen planus (LP) is a chronic inflammatory disease of the skin and mucous membranes [1], occurring primarily in people aged between 30 and 60 years with an increased incidence in females [2]. The prevalence of oral lesions ranges from 0.1 to 2.2% [3]. Lichen lesions on the skin are self-limiting, while oral mucosal lesions are chronic and undergo numerous phases of remission and exacerbation. Clinical forms of OLP vary from reticular, a mostly asymptomatic form, characterized by the appearance of hyperkeratotic Wickham’s striae, to erosive, which causes pain and discomfort due to the appearance of erosions [4]. The etiology of OLP has not been fully elucidated, and available studies in the literature emphasize its immunopathogenic basis with enhanced cytokine regulation [5].

The possible association of OLP and psychological disorders such as anxiety, depression, and stress has been documented in the literature [6]. A study of Di Stasio et al. [6] evaluated the prevalence of psychiatric symptoms in 11 OLP patients compared to 13 controls. In the OLP group, 73% of patients presented with a VAS score of a mild type, 9% had depressive symptoms, and 100% had a score above the cut-off for anxiety; however, no statistically significant difference was found when compared to the control group. The authors concluded that a larger sample size could possibly lead to different results. Furthermore, there are data from the literature on a rare type of lichen planus called vulvovaginal gingival lichen planus (VVG-LP) [7]. Approximately 20% of women with OLP develop vulvovaginal lesions [7]. Lucchese et al. [8] reported two cases of VVG-LP and pointed out the importance of a multidisciplinary approach requiring an accurate diagnosis as well as a regular check-up of oral and genital lesions due to the possible development of carcinoma in the affected mucous membranes [9]. 

The World Health Organization has categorized OLP as a potentially malignant disorder [10]. According to a recent systematic review, the malignant transformation rate for OLP is 1.37% [11].

OLP lesions are treated in the acute phase of the disease, i.e., when there is inflammation and/or epithelial discontinuity accompanied by symptoms of pain and discomfort. The most commonly administered drugs are topical corticosteroids [12]. In addition to topical administration, systemic corticosteroids and perilesional corticosteroid injections can be used in severe cases [13,14]. Administering systemic corticosteroids requires caution due to a number of serious side effects when administered over a long period of time and in large doses [13,15]. Retinoids and immunosuppressants are the drugs most commonly used with corticosteroids in treating OLP, but with caution due to numerous side effects and frequent relapses. In recent years, new therapeutic methods have emerged, such as dapsone, antimalarials, interferon, hyaluronic acid, PUVA therapy, and many others, but with limited application and efficacy; therefore, additional clinical trials are necessary [16].

The purpose of this paper was to present current therapeutic options in the treatment of OLP and gain insight into their effectiveness and safety in treating OLP lesions. The review covers all the therapeutic options for the treatment of OLP that are mentioned in the literature. PubMed was used in the literature search, and studies published in the period from 1970 to 2022 were included.

## 2. Treatment of OLP

Since OLP is a disease of unknown etiology, treatment is symptomatic and involves the suppression of signs and symptoms of the disease. Various topical and systemic drugs are used for this purpose.

Potent topical corticosteroids have been accepted as first-line treatment; however, no solid scientific evidence supports this fact. A lack of convincing evidence regarding the efficacy of previous therapies, including topical corticosteroids accepted as first-line OLP treatment, has been noted in the Cochrane systematic review [17]. The reasoning behind this is that the authors note the small number of included studies and participants. Today, topical or perilesional administration of corticosteroids is most commonly used as first-line therapy in the treatment of the erosive form of OLP [12]. However, there is no clinical agreement on second-line therapy, although short-term use of systemic corticosteroids is used to rapidly control the signs and symptoms of the disease, or treat persistent lesions that do not respond to topical steroid therapy [17].

Since the erosive form of OLP causes unpleasant symptoms of pain, thus limiting the quality of life of patients, the goal of treatment is to use the most effective therapeutic preparation. In addition to corticosteroids, numerous studies in the literature have examined the effectiveness of various drugs and preparations in the treatment of OLP [18]. A list of therapeutic options discussed in the article is shown in Table 1.

### 2.1. Corticosteroids

Synthetic corticosteroids are produced based on the chemical structure of the glucocorticoid hydrocortisone (cortisol). Cortisol, as the main representative of glucocorticoids, affects the metabolism involving carbohydrates, proteins and fats, as well as having anti-inflammatory and immunosuppressive effects [19]. In addition to being a replacement therapy for adrenal cortex insufficiency, corticosteroids can also be used in various inflammatory diseases of unknown etiology (rheumatic diseases, lupus, sarcoidosis...), severe allergic diseases, and in dentistry for the treatment of mucosal lesions of different etiologies (OLP, pemphigus, traumatic ulcer) [20].

#### 2.1.1. Topical Corticosteroids

Today, in the treatment of OLP, topical steroid preparations are most often used in the form of ointments, gels, creams, adhesive pastes, rinsing solutions and sprays. In the majority of cases, OLP lesions can be controlled with high-potency topical steroid preparations, which have been proven to be very effective and have a lower incidence of side effects than systemic corticosteroids. Topical corticosteroids can be divided according to potency (Table 2) [21]. 

Triamcinolone acetonide is a topical corticosteroid used in the form of an adhesive paste, lozenge or oral suspension in the treatment of OLP [22]. Solutions of betamethasone disodium phosphate and clobetasone propionate are successfully used as a therapy for the diffuse form of OLP, with an increased possibility of systemic absorption and complications caused by adrenal gland suppression [23]. Betamethasone valerate, clobetasol, fluocinonide acetonide, fluocinonide and triamcinolone acetonide adhesive paste are often used in the treatment of OLP [22,24,25,26,27], whereas fluticasone propionate is used as a short-term treatment due to its low tolerance [28]. Generally, 0.05% fluocinonide and 0.5% fluocinolone acetonide are used in more severe, stubborn cases that do not respond successfully to therapy [22,27], including highly potent clobetasone due to its effectiveness in maintaining disease remission [24,25]. 

Oral candida infection is the most common side effect of topical corticosteroids, and can be prevented by using antifungals and chlorhexidine mouthwash solutions [22,24,27]. An intralesional corticosteroid injection provides the desired therapeutic effect quickly; however, the presence of pain and discomfort and the possibility of developing atrophy at the site of application reduces the popularity of this approach [14]. Topical therapy is indicated for localized lesions with a minimal possibility of systemic absorption and undesirable effects on the adrenal gland. According to the literature, in addition to local side effects in the form of candidiasis and atrophy [14], data can be found on side effects associated with systemic absorption (hirsutism, moon face between the fourth and sixth week of topical therapy) [23]. Other side effects that occur less frequently are dry mouth, bad taste and smell, swollen lips, nausea [28], two cases of hairy leukoplakia in immunocompetent patients [24], hypersensitivity reaction of the oral mucosa [29], hemorrhagic effusions on the skin and mucous membranes when administering 0.05% clobetasol propionate solution [30] and iatrogenic Cushing’s syndrome in patients with OLP and pemphigoid with 0.05% clobetasol propionate therapy [31].

#### 2.1.2. Systemic Corticosteroids

Systemic corticosteroids are considered the most effective in treating patients with diffuse erosive OLP, in those cases where the disease does not respond to topical steroid preparations, and in patients with the mucocutaneous form of the disease. For this purpose, methylprednisolone and prednisone are prescribed in doses of 1.5–2 mg/kg/day, gradually lowering the dose after clinical improvement. Systemic administration of corticosteroids has significantly more serious and frequent side effects than topical preparations; hence caution is required when prescribing them [13,15,32].

Numerous serious side effects are associated with the systemic use of corticosteroids, and their appearance depends on the dose and duration of therapy. The most serious complication that occurs due to the suppression of the adrenal gland caused by corticosteroids is an adrenal crisis. Systemic administration of corticosteroids has numerous other side effects, the most significant of which is iatrogenic Cushing’s syndrome, characterized by sodium and water retention, hypokalemia, hyperlipidemia, obesity in the central part of the body and moon face. Osteoporosis, hyperglycemia, diabetes, hypertension, glaucoma, cataracta, atrophic skin changes with purpura, slow wound healing, a tendency to easily bruise, gastritis, ulcers, catabolic effects on skeletal muscles and loss of muscle mass, immunosuppression, and psychiatric symptoms can also occur [33].

### 2.2. Immunosuppressants

Immunosuppressive drugs inhibit the proliferation and functioning of T lymphocytes and lead to a decrease in the immune response of the body [34]. In the treatment of OLP, cyclosporine, azathioprine, tacrolimus and pimecrolimus are used. Although many studies have confirmed the effectiveness of immunosuppressants in the treatment of OLP, the side effects such as bad taste, burning sensations after the first application, nephrotoxicity, hypertension and the high price of the drug should be pointed out [35,36,37].

#### 2.2.1. Cyclosporine (CSA)

Cyclosporine (CSA) is a macrolide immunosuppressant drug, primarily indicated for the prevention of transplant rejection with the aim of suppressing the patient’s immune system. CSA reversibly and specifically suppresses T-lymphocyte activity. By binding to the intracellular protein cyclophilin, found in lymphocytes, CSA forms a complex of CSA–cyclophilin that further inhibits calcineurin. Calcineurin is a protein that, under normal conditions, induces the transcription of interleukin IL-2, and its inhibition reduces the amount of IL-2 and reduces the functioning of T-lymphocytes. CSA, along with tacrolimus and pimecrolimus, is also a calcineurin inhibitor [34]. Due to its specific action, it is used in the treatment of stubborn OLP cases as mouthwash (100 mg per ml, two times daily) or topically in an adhesive paste. The disadvantages are its high price, hydrophobicity, unpleasant taste, and cyclosporine-induced gingival hyperplasia [5].

#### 2.2.2. Tacrolimus and Pimecrolimus

Tacrolimus (FK-506) is a macrolide immunosuppressant from the group of calcineurin inhibitors and is used to prevent organ rejection. Compared to CSA, tacrolimus has a greater capacity for penetration through the mucosa and 10–100 times greater potency [34]. In the treatment of OLP, it is applied topically in a concentration of 0.1% to the affected area twice a day. It has been proven to be effective in the treatment of erosive OLP but with certain side effects in the form of mucosal burning and relapse within 12 months of ceasing the therapy. Pimecrolimus has a similar effect to tacrolimus and is used topically in a concentration of 1% in the treatment of OLP [5,38].

#### 2.2.3. Azathioprine

Azathioprine (AZA) is an antimetabolite that disrupts purine synthesis and leads to reduced proliferation of T and B lymphocytes [34]. In addition to its immunosuppressive action, it also has important anti-inflammatory properties. AZA is used in generalized cases, systemically and with great caution due to numerous serious side effects such as bone marrow suppression, pancytopenia and liver dysfunction [39].

### 2.3. Retinoids

Retinoids are metabolites of vitamin A (rethionol) and are used topically or systemically in the treatment of OLP. In the body, they are required for the normal growth and proliferation of epithelial cells, and in case of deficiency, epithelial cells in the oral cavity become keratinized [40]. The most commonly used topical retinoids are tretinoin, isotretinoin and fenretinide in a gel form (0.1%). The use of these topical retinoids leads to a reduction of reticular and plaque lesions, as well as a recurrence after discontinuing therapy [41]. In terms of systemic retinoids, the most common are etretinate, isotretinoin and tretinoin, and their use in the treatment of OLP is restricted due to numerous side effects such as cheilitis, liver damage and a teratogenic effect. Temaroten has been shown to be effective in treating OLP, resulting in fewer side effects [5,41].

### 2.4. Other Drugs and Preparations

#### 2.4.1. Dapsone

Dapsone has shown modest results in treating the erosive form of OLP [42,43], and considering the side effects, such as hemolysis and headache associated with its use [44], it is not the drug of choice for OLP.

#### 2.4.2. Amlexanox

Amlexanox (C16H14N2O4) is a topical anti-inflammatory drug approved for the treatment of recurrent aphthous ulcers [45]. Its action is based on the ability to inhibit the formation and release of histamine, TNF-α, leukotrienes from mast cells, neutrophils and mononuclear cells [46]. So far, only a single pilot study has evaluated the effectiveness of topically applied amlexanox in the form of a paste in treating erosive OLP. The results of the study show that there was no statistically significant difference between amlexanox and dexamethasone (control group) in reducing the clinical signs and symptoms of the disease. The authors suggest applying amlexanox topically as a possible alternative to topical corticosteroids. Side effects of the topical application of amlexanox, such as burning and dry mouth, as well as bleeding at the site of applying the drug, have also been recorded [47].

#### 2.4.3. PUVA Therapy

PUVA therapy or photochemotherapy, requiring local or systemic application of the photosensitizer psoralen, is successfully used in the treatment of the skin LP [48,49], while it was used in OLP for the first time in a resistant case [50]. A significant improvement in 87% of OLP patients treated with UVA rays without systemic or topical photosensitizers was observed [51]. The results of a controlled study showed the effectiveness of PUVA therapy with systemic administration of psoralen in the treatment of severe forms of OLP [52]. Side effects of PUVA therapy include nausea, dizziness, ocular symptoms, headache and paresthesia [53]. In addition to the mentioned side effects, PUVA therapy is also associated with an oncogenic potential [53], and therefore is recommended in the treatment of more severe forms of OLP that do not respond to conventional therapy [54].

#### 2.4.4. Antibiotics 

Tetracyclines were used in the treatment of OLP; however, their effectiveness is limited to a few cases of gingival lesions of OLP [55,56]. Today, antibiotics are not recommended for routine treatment of OLP [56].

#### 2.4.5. Antimalarial Drugs 

Hydroxychloroquine sulfate has shown clinical efficacy in 9 out of 10 OLP patients [57], as well as three-month therapy with chloroquine phosphate in patients with LP lesions on the lower lip [58]. Considering the possibility of developing a lichenoid reaction due to the use of antimalarials, they are not the treatment of choice for OLP [59].

#### 2.4.6. Glycyrrhizin 

Licorice is a plant that has been used in medicine for centuries. Glycyrrhizin, found in part of the root structure of licorice, has been successfully used in the treatment of OLP in patients with chronic hepatitis C [60,61]. Given the hepatoprotective effect of glycyrrhizin, its efficacy in the treatment of OLP lesions needs to be further investigated.

#### 2.4.7. Interferon 

In the treatment of erosive OLP, topical application of alpha and beta-interferon has been suggested [62]. The development and exacerbation of OLP have been observed during and after alpha-interferon therapy in HCV patients [63,64,65], although systemic alpha-interferon has been successfully used in the treatment of OLP in patients with and without HCV infection [66,67,68].

#### 2.4.8. Levamisole 

Levamisole has been used as an immunomodulator in the treatment of OLP [69]. A prospective study suggests a combined therapy of low doses of systemic corticosteroids and levamisole controlling the more severe erosive form of OLP [70]. However, the appearance of lichenoid lesions on the skin and in the oral cavity of patients who used levamisole in the treatment of rheumatoid arthritis has also been noted. The mentioned changes regressed upon the discontinuation of levamisole [71].

#### 2.4.9. Mesalazine 

Mesalazine or 5-aminosalicylic acid is a drug used in the treatment of inflammatory bowel diseases. The use of topical mesalazine in the treatment of symptomatic OLP has been shown to be as effective as topical clobetasol [72]. As with most preparations for the treatment of OLP mentioned so far, the appearance of lichenoid lesions is also associated with the use of mesalazine [73].

#### 2.4.10. Phenytoin 

A study in the literature reported the use of phenytoin in the treatment of OLP. The results showed complete healing in two out of four OLP patients treated with phenytoin [74]. Further research did not confirm the effectiveness of phenytoin in the treatment of OLP, nor the occurrence of unwanted effects after the applied therapy; however, it is known that phenytoin can also lead to the appearance of lichenoid lesions [75].

#### 2.4.11. Aloe Vera 

The use of aloe vera in the treatment of OLP has been described in four studies. The effectiveness of aloe vera was described in a study in which it was applied as a topical and systemic therapy [76]. A randomized, double-blind, placebo-controlled study evaluating the effectiveness of topically applied aloe vera gel showed a statistically significant improvement in clinical signs of the disease and a reduction in subjective complaints compared to the placebo [77]. The use of aloe vera in the form of a solution during four weeks of therapy has been shown to be effective in treating clinical signs and symptoms of OLP [78]. A double-blind, randomized study showed the effectiveness of aloe vera gel in improving the quality of life of patients with OLP compared to placebo [79].

#### 2.4.12. Monoclonal Antibodies 

The results of a prospective (open-label) pilot study suggest that the monoclonal antibody efalizumab is a potential therapy for erosive OLP and the ulcerative skin form of the disease. However, two of the four subjects developed serious side effects during treatment with efalizumab. One of the subjects was hospitalized due to urticaria and a staphylococcal infection of the hip, while the other patient developed subacute cutaneous lupus. Therefore, the authors suggest particular caution when prescribing this drug [80].

#### 2.4.13. Sulodexide 

Sulodexide is a drug with a low molecular weight, made of heparin chains (80%) and dermatan sulfate (20%), with an extremely low anticoagulant effect. The therapeutic effect of sulodexide is related to the action of plasmin, cell proliferation and cytokine activation [81,82]. Also, due to its protective effect on the endothelium [83] and helping repair cellular damage [82,84], sulodexide may have a potential role in treating and controlling the erosive form of OLP. An open, non-randomized study comparing the efficacy of systemic sulodexide and topical cyclosporine was conducted on a total of twenty patients with chronic erosive OLP, whose lesions did not respond to topical corticosteroid therapy. Control of pain and reduction of clinical signs of the disease was recorded in both groups of subjects; however, there was slightly faster healing in the group of subjects who were treated with systemic sulodexide. Side effects were noted in a subject treated with sulodexide. Due to dizziness and vomiting during the second day of therapy, the subject was excluded from the study [85].

#### 2.4.14. Reflexo-Therapy 

The use of reflexology in the treatment of symptomatic OLP is also described in the literature. The authors propose several treatment models that accelerate the epithelization of erosive lesions and ulcerations on the buccal mucosa and emphasize the strong analgesic effect of the applied therapy [86].

#### 2.4.15. Surgery 

Surgical excision [87,88] and cryosurgery [89] have been used successfully in cases of erosive OLP when resistant to most therapeutic options [90]. After removing lesions by cryosurgery, relapse is common [91], as well as the formation of new lesions in healing wounds and on scars, accompanied by stronger symptoms. Free soft tissue graft [92] and free gingival tissue graft [93] have also been used in the treatment of localized lesions of erosive OLP.

#### 2.4.16. Hyaluronic Acid

Topical hyaluronic (HA) acid was used in the treatment of OLP lesions. A randomized, placebo-controlled, double-blind study evaluated the efficacy of topical HA gel preparation (0.2%) in patients with erosive OLP. Results of the study showed a significant reduction in soreness scores when compared to the placebo for up to 4 h post-application. Additionally, a significant reduction in the size of the lesion after 28 days of treatment when compared with baseline was detected, but with no significant difference in lesion size between the treatment groups. The authors suggest topical HA gel as a useful addition to the treatment option for OLP with frequent application [94]. Another study compared the efficacy of a triamcinolone (TA) preparation (0.1%) with an HA preparation (0.2%) in the management of 40 OLP patients. The results showed improvement in pain score and lesion size, with no difference between the treatment groups [95]. 

#### 2.4.17. Curcumin

Curcuma longa is a plant that has been used for centuries in traditional Indian medicine due to its anti-inflammatory effects [96]. The anti-inflammatory, antioxidant, anticarcinogenic effects of improving the healing of wounds as well as the safety of curcumin have been documented [97].

The study by Kia et al. in 2015 compared the efficacy of 5% curcumin and 0.1% triamcinolone in the form of an oral paste applied three times a day over the course of four weeks [98]. Nine patients (36%) in the curcumin group and eight patients (32%) in the triamcinolone group experienced complete pain reduction. One patient (4%) in each group showed complete remission of the lesion score. No statistically significant difference was noted between the two groups. A few patients in the curcumin group had complained of a burning sensation, swelling and xerostomia, which disappeared at the end of the first week of therapy. The majority of patients complained of yellow-colored gingiva. In the triamcinolone group, one patient complained of a burning sensation in the first week of therapy, and one patient complained of mucosal desquamation during the four weeks of therapy. The authors suggest the use of topical curcumin as an alternative to synthetic drugs for OLP treatment due to its desirable anti-inflammatory effects and insignificant side effects [98]. Furthermore, the authors of another study proposed curcumin as a maintenance drug after the patient is treated with corticosteroids [99]. In the study by Amirchaghmaghi et al. [100], no effect of curcumin in the treatment of OLP was detected.

#### 2.4.18. Vitamin D

Considering that vitamin D receptors (VDRs) are expressed on cells involved in the immune system, such as T and B cells, this indicates its crucial role in immunity [101]. A pilot study on the efficacy of vitamin D supplementation in the treatment of OLP lesions showed statistically significant improvement in subjective and objective symptoms in patients who were supplemented with vitamin D with or without psychological counseling apart from topical steroid application for a short period. The authors emphasized the small sample size and uneven distribution of subjects due to the dropout of cases. However, they pointed out the possible role of vitamin D in pathogenesis and, consequently, the treatment of OLP [102]. In the study undertaken by Nazeer et al. [103], 450 OLP patients were divided into three groups according to their vitamin D levels. Maximum improvements were detected in patients who were given vitamin D supplementation. 

#### 2.4.19. Selenium

Selenium (Se), an essential trace element with an antioxidant effect found naturally in the human body, acts against oxidative stress, slows down the aging process, and inhibits viral infections while playing an important role in chemoprevention, metabolism and immune system modulation [104]. In a randomized controlled clinical trial, two Se forms (novel topical hydrogel and oral capsules) in treating erosive OLP were evaluated. Patients were allocated into three groups: group I—topical corticosteroids; group II—topical Se; and group III—systemic Se. Treatment lasted for 6 weeks. A significant reduction of signs and symptoms in response to all treatment modalities was detected. However, there was no significant difference between the three groups in the sixth week of therapy. Compared to topical corticosteroids, selenium showed some advantages, such as a longer persistent effect, good pain reduction, and no increased risk of developing a secondary infection [105]. However, further clinical trials with larger sample sizes are necessary to confirm these results. 

#### 2.4.20. NAVS Naphthalan

Non-aromatic very rich in steranes (NAVS) naphthalan is an earth mineral oil containing similar chemical structures to vitamin D3 and steroid hormones [106,107]. The effectiveness of NAVS in the treatment of OLP was evaluated in two studies. A pilot study demonstrated the reduction of clinical signs and symptoms in 11 OLP patients [108]. In a double-blind, randomized, controlled clinical trial, 39 OLP patients received either NAVS or 0.05% betamethasone dipropionate oral paste for 28 days. Patients in both groups showed improvements, but the results failed to show any significant differences according to the activity score of the disease and the intensity of pain between the groups [109]. NAVS did not show any side effects compared to the betamethasone group, where three patients developed a candida infection. Given the small sample size of this study, further clinical trials are needed. 

#### 2.4.21. Photodynamic Therapy (PDT)

Photodynamic therapy (PDT) is an alternative treatment option for OLP. It is based on the interaction between a light source and the administration of a photosensitizer (PS). This interaction produces free radicals and leads to cell damage [110]. Methylene blue (MB), 5-aminolevulinic acid (5-ALA) and chlorin e6 derivative can be used as a PS. The efficacy of PDT in the treatment of OLP evaluated in a systematic review and topical use of 5-ALA showed a better response than MB [111]. Having searched for studies in the literature, 16 studies were included in the qualitative analysis and 13 in the quantitative analysis. The authors concluded that PDT is as effective as topical corticosteroids and can be used for OLP resistant to corticosteroids or in cases when they are contraindicated. Certain limitations exist, such as the insufficient number of studies meeting the inclusion criteria, different outcome measures and heterogeneity for wavelength and energy density. For these reasons, more high-quality studies are necessary to confirm the obtained results [111]. 

#### 2.4.22. Low-Level Laser Therapy (LLLT)

Low-level laser therapy (LLLT) has been proposed as a treatment option for OLP. Helium–neon, ultraviolet and diode lasers with different output powers, doses, irradiation times and the number of sessions are used in the treatment of OLP lesions [112,113]. Photobiomodulation (PBM) (at 400–1.100 nm wavelength) has a beneficial effect on cell metabolism without damaging cells or the basal temperature of the body [114]. Nonetheless, there is no generally accepted standardized clinical protocol in 2021. Del Vecchio et al. [114] proposed doses oscillating between 2 and 3 J/cm^2^ as an effective treatment of OLP lesions. The effectiveness of LLLT in the treatment of erosive OLP in 42 year-old male patient has been reported [115]. The lesion was treated with a 660 nm diode laser for two sessions (5 min each session), once a week. After two weeks, the lesion completely healed. No relapse during four months of follow-up period was reported [115]. 

In 2018, a systematic review by Akram et al. [116] evaluated the efficacy of LLLT compared to corticosteroid therapy in treating OLP. It included five clinical studies with laser wavelengths between 630 and 970 nm, at a power of 10.3000 mW, a spot size of 0.2–1.0 cm^2^ and laser exposure of 6–480 s. Three studies showed a significantly higher improvement using topical corticosteroids compared to LLLT. Only one study using LLLT showed significant improvement of the OLP lesions, with one study showing results comparable to LLLT and topical corticosteroid treatment. The authors emphasized a high risk of bias in four out of five clinical studies and concluded that it remains uncertain whether LLLT is more effective for the treatment of OLP when compared to corticosteroids due to weak scientific evidence [116]. The purpose of another recently published systematic review was to determine the efficacy of PBM in the treatment of atrophic–erosive OLP compared to other treatments [117]. After identifying a total of 297 studies, 7 clinical studies met the inclusion criteria and were included in the systematic review. Most of the studies used topical corticosteroids as a control group, while some studies compared PBM with another type of laser plus corticosteroids [117] or with cold knife surgery [118]. The results showed that PBM was good in reducing the signs and symptoms caused by atrophic–erosive OLP. A study by Mutafchieva et al. [119] showed clinical improvements in 59.3% of the OLP lesions, whereas a study by Khater et al. [120] showed complete remission in 37.3% of cases. The limitations of this systematic review are the small sample size, lack of follow-up, high heterogeneity in the characteristics of the control groups, different methodologies and analyzed parameters to assess the effectiveness of PBM [121]. The authors concluded that only a generally accepted standardized clinical protocol would be of great clinical benefit for future randomized clinical trials for the purpose of obtaining strong scientific evidence. 

## 3. Discussion

Lichen planus (LP) is a common chronic immune inflammatory disease that affects skin and mucous membranes [122]. Due to its unknown etiology, the treatment is symptomatic and involves suppressing signs and symptoms of the disease. The lack of convincing evidence on the effectiveness of many therapies, including topical corticosteroids, was noted in a Cochrane systematic review [17]. Upon searching the database, a total of two hundred and twenty articles were identified, addressing the subject of the current therapeutic possibilities of OLP. The systematic review included fifteen randomized studies, with a total of six hundred and sixty-seven subjects, of which four hundred and seventy-three had the erosive form of OLP. Most of the studies had a small number of subjects (from twelve to thirty-nine). Furthermore, the vast majority of the studies were heterogeneous in terms of using different outcome variables and the clinical score of disease. Therefore, the authors of the Cochrane systematic review suggest using standardized methods for evaluating the outcomes of treatment and assessing the clinical score of the disease. In six studies, patients with a non-erosive form of OLP were also included; however, only patients with erosive OLP were included in this systematic review. In the end, it was not possible to combine the results of the conducted studies, given the mentioned heterogeneity [17]. An update of the mentioned review was published in 2020 and focused on the evidence solely for corticosteroid therapy [123]. The aim of this systematic review was to assess the effectiveness and safety of any corticosteroid therapy for symptomatic OLP. The systematic review included 35 randomized trials, 7 studies at low risk of bias overall, 11 unclear and the remaining 17 studies at high risk of bias. The results showed that topical corticosteroids in an adhesive paste are effective in controlling pain in OLP, but evidence of this finding is low. Furthermore, there is no convincing evidence that one corticosteroid therapy is better or worse than another. There is also no convincing evidence that calcineurin inhibitors, such as tacrolimus, are more effective in reducing pain than corticosteroids [123]. It seems that the effectiveness of the topical application of corticosteroids is the most frequently investigated therapeutic option in the treatment of OLP, either as a test or control group in the studies published so far. Furthermore, considering the previously mentioned therapeutic options in the treatment of OLP, it seems that topical corticosteroids are widely used as a first-line therapy due to their effectiveness in the treatment of inflammation and pain with minimal side effects. Additionally, the cost–benefit of using them should not be ignored.

## 4. Conclusions

Despite having a very good understanding of the immunopathogenic mechanism of OLP, the trigger that initially activates the formation of the lesions remains unknown. Therefore, it is not surprising that there is currently no ideal therapeutic agent in the treatment of OLP, which can be concluded from the previously mentioned wide range of therapeutic options. All offered treatment methods only partially satisfy the properties of effectiveness and safety of application, which justifies the continuous search and testing of new preparations and methods of treatment.

## Figures and Tables

**Table 1:** Therapeutic options in the treatment of OLP.

Corticosteroids	Retinoids	Immunosuppressants	Other Drugs and Preparations
Topical: betamethasone phosphate, clobetasol propionate, flucinolone acetonide, flucinonide, fluticasone propionate, hydrocortisone hemisuccinate, triamcinolone acetonide Systemic: prednisone, methylprednisolone	Topical: fenretinide, isotretinoin, tazaroten, tretinoin Systemic: acitretin, etretinate, isotretinoin, temarotene, tretinoin	azathioprine, cyclosporine, pimecrolimus, tacrolimus	basiliximab, dapsone, doxycycline, glycyrrhizin, hydroxychloroquine sulphate, interferon, levamisole, mesalazine, phenytoin, PUVA, reflexology, surgery, thalidomide, aloe vera, amlexanox, alefacept, efalizumab, sulodexide, hyaluronic acid, curcumin, vitamin D, selenium, NAVS, photodynamic therapy, Low Level Laser Therapy (LLLT)

**Table 2:** Topical steroid preparations according to potency.

Low potency corticosteroids	1% hydrocortisone acetate 0.05% alclomethasone dipropionate 0.25% methylprednisolone acetate
Medium potency corticosteroids	0.05% clobetasone butyrate 0.1% hydrocortisone butyrate 0.5% fluocortolone pivalate
Highly potent corticosteroids	0.025% beclomethasone dipropionate 0.05% beclomethasone dipropionate 0.025% betamethasone benzoate 0.1% betamethasone valerate 0.1% diflucortolone valerate 0.025% flucinolone acetonide 0.05% fluticasone propionate 0.05% flucinonide
Very highly potent corticosteroids	0.05% clobetasol propionate 0.3% diflucortolone valerate 0.01% halcinonide

## Data Availability

Not applicable.

## Abbreviation

OLP	Oral lichen planus
LP	Lichen planus
VVG-LP	Vulvovaginal gingival lichen planus
VAS	Visual analogue scale
PUVA	Photochemotherapy
CSA	Cyclosporine
IL	Interleukin
AZA	Azathioprine
HCV	Hepatitis C virus
HA	Hyaluronic acid
Se	Selenium
NAVS naphthalan	Non-aromatic very rich in steranes naphthalan
PDT	Photodynamic therapy
MB	Methylene blue
5-ALA	5-aminolevulinic acid
LLLT	Low level laser therapy
PBM	Photobiomodulation
